# The role of p38b MAPK in age-related modulation of intestinal stem cell proliferation and differentiation in Drosophila

**DOI:** 10.18632/aging.100054

**Published:** 2009-05-21

**Authors:** Joung-Sun Park, Young-Shin Kim, Mi-Ae Yoo

**Affiliations:** Department of Molecular Biology, Pusan National University, Busan 609-735, Korea

**Keywords:** Drosophila, p38b MAPK; aging, oxidative stress, intestinal stem cell, gut, PVR signaling, Delta/Notch pathway, proliferation, differentiation

## Abstract

It is important to understand how age-related changes in intestinal stem cells (ISCs) may contribute to age-associated intestinal diseases, including cancer. *Drosophila* midgut is an excellent model system for the study of ISC proliferation and differentiation. Recently, age-related changes in the Drosophila midgut have been shown to include an increase in ISC proliferation and accumulation of mis-differentiated ISC daughter cells. Here, we show that the p38b MAPK pathway contributes to the age-related changes in ISC and progenitor cells in *Drosophila*. D-p38b MAPK is required for an age-related increase of ISC proliferation. In addition, this pathway is involved in age and oxidative stress-associated mis-differentiation of enterocytes and upregulation of Delta, a Notch receptor ligand. Furthermore, we also show that D-p38b acts downstream of PVF2/PVR signaling in these age-related changes. Taken together, our findings suggest that p38 MAPK plays a crucial role in the balance between ISC proliferation and proper differentiation in the adult *Drosophila* midgut.

## Introduction

In mammals, intestinal homeostasis is maintained by the balance between intestinal stem cell (ISC) proliferation, directed differentiation, and removal of dead cells in adults [1]. The precise mechanism by which proliferation and differentiation of stem cells is lost with age and/or oxidative stress is unknown. These effects on stem cells result in age-related diseases, such as cancer [2,3]. Therefore, it is important to understand how age-related changes in ISC proliferation and differentiation contribute to age-associated intestinal diseases, including cancer. Recently, *Drosophila melanogaster* was shown to be an excellent model system for the study of ISC biology and aging. It was demonstrated that proliferating progenitor cells reside within the intestinal epithelium of adult *Drosophila*, similar to vertebrate intestine [4,5]. Adult *Drosophila* midgut cells can be identified as ISCs, enteroblasts (EBs),  enteroendocrine cells (EEs) and enterocytes (ECs) with specific markers. ISCs can be identified by the expression of Delta, a Notch receptor ligand [6]. *Escargot *(*esg*) is a marker for ISCs and EBs [5]. Prospero is a marker for EEs [4,5]. The *Su(H)GBE-lacZ* reporter construct is induced by Notch activity. Therefore, expression of lacZ is a marker for EBs, since the fates of ISC daughter cells are specified via differential Notch activity modulated by expression levels of Delta in ISCs [6]. Previously, we reported age-related increases in ISC proliferation and in the number of Delta-/esg-/Su(H)GBE-positive cells [7]. We also showed that oxidative stress can mimic age-related changes in ISCs and that a PDGF/VEGF-like growth factor, PVF2/PVR, is involved in age and oxidative stress-related changes [7]. Biteau et al. reported that this increase in the number of Delta-/esg-/Su(H)GBE-positive cells is due to the accumulation of mis-differentiated ISC daughter cells such as EC-like large esg-positive cells. In addition they showed that the age-related changes are associated with aberrant Delta/Notch and JNK signaling [8]. More recently, involvement of the insulin receptor and the JAK-STAT signaling pathways was shown in ISC proliferation in response to tissue damage and during immune response, respectively [9,10].

In mammals, it has been well demonstrated that p38 MAPK is activated in response to various physical and chemical stresses, such as oxidative stress, UV irradiation, hypoxia and ischemia [11]. Fu et al. reported increased p38 MAPK activation in ISCs and their daughter cells in the small intestine after ischemia [12]. In *Drosophila*, two p38 MAPK isoforms have been identified, D-p38a and D-p38b. D-p38s are activated by various stresses, including UV, lipopoly-saccharide (LPS), and osmotic stress [13,14]. It was reported that D-p38b mRNA levels were detected in the developing posterior midgut [14]. We previously observed an increase in expression of a *D-p38b-lacZ* reporter construct in the posterior midgut by septic injury [15]. However, the role of p38 MAPK in ISC proliferation and differentiation remains unknown.

**Figure 1.**
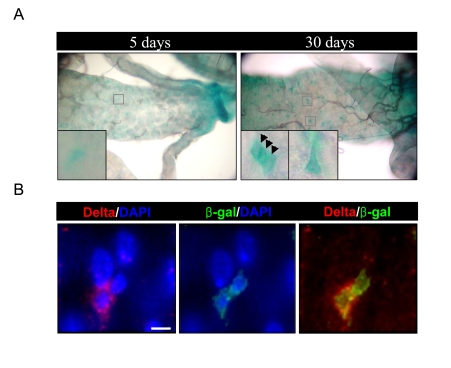
Increased expression of D-p38b in ISCs and EBs within aged gut. (**A**) Increased expression of *D-p38b-lacZ* reporter construct in the adult posterior midgut with age. The midguts of 5- and 30-day-old flies, including those with two copies of the *D-p38b-lacZ,* were examined by X-gal staining. Squared boxes are enlarged images. Arrow head indicates β-gal-positive cells. Original magnification is 400x. (**B**) Increased expression of *D-p38b-lacZ* reporter construct in the Delta-positive and neighboring cells in aged gut. The midguts of 30-day-old flies were examined with anti-β-gal and anti-Delta. Anti-β-gal, green; anti-Delta, red; DAPI, blue. Scale bar, 1 μM.

In the current study, we tested whether D-p38b MAPK is involved in age and oxidative stress-associated modulation of ISC proliferation and differentiation in the adult *Drosophila* midgut. We further examined the serial relationship between D-p38b MAPK and PVR signaling in age-associated intestinal changes.

## Results

### Increased expression of a *D-p38b-lacZ* reporter construct in ISCs and progenitors of aged midgut

To determine the role of D-p38b MAPK in age-related changes in the adult *Drosophila* midgut, we first investigated D-p38b expression in the adult midgut using transgenic flies carrying a *D-p38b-lacZ* reporter construct [15]. Increased expression of the *D-p38b-lacZ* reporter construct in aged midgut was detected by X-gal staining (Figure 1A). In Figure 1B, we analyzed the expression pattern of* D-p38b-lacZ* in the aged guts using anti-β-gal antibody and anti-Delta antibody, a marker of ISC [4,5]. Interestingly, the expression pattern of *D-p38b-lacZ* in young and aged posterior midguts indicates that the expression of *D-p38b-lacZ* increases in ISCs and neighboring cells in the adult *Drosophila* midgut.

**Figure 2.**
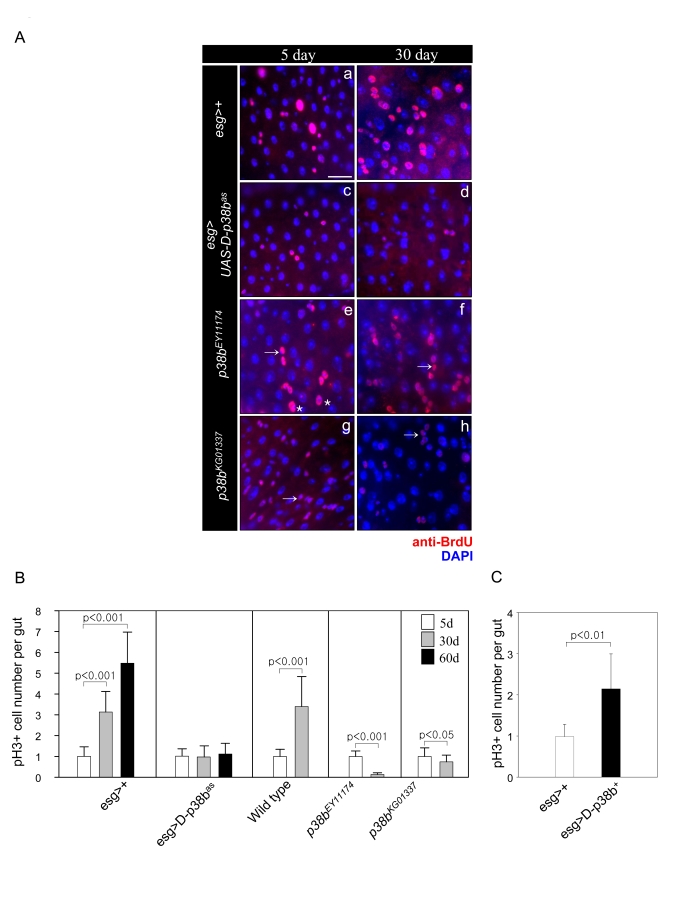
Effect of D-p38b MAPK signaling on DNA synthesis of intestinal cells and ISC division. (**A**) Effects of D-p38b MAPK modulation on BrdU incorporation levels in the adult midgut. Twenty-five day-old flies expressing *esg>+ *(a and b),* esg>UAS-D-p38b^as^*(c and b), *p38b^EY11174 ^*(e and f) or *p38b^KG01337^* (g and h) were fed on 0.2 mg/ml BrdU media for 4 days, and stained with anti-BrdU. Overlay (DAPI, blue; anti-BrdU, red). Asterisk indicates enlarged EC nuclei. Arrow indicates small ISC, EB or EE cell nuclei. Scale bar, 5 μM. Original magnification is 400x. (**B**) Effect of D-p38b MAPK activity on the number of PH3-positive cells within the adult gut. Number of PH3-positive cells detected per midgut of 5-, 30- and 60-day-old *esg>+* or* esg>UAS-D-p38b^as^* flies and 3- and 30-day-old control flies, *p38b^EY11174^* or *p38b^KG01337^*. The number of PH3-positive cells detected per midgut of 5-day-old flies was set as 1. White bar, 5-day-old flies; gray bar, 30-day-old flies; black bar, 60-day-old flies. P-values were calculated using Student's t-test. (**C**) Effect of D-p38b MAPK activation on the number of PH3-positive cells. Number of PH3-positive cells in the midguts of 5-day-old flies carrying *esg>+* or* esg>UAS-D-p38b^+^* were analyzed. White bar, *esg>+*; gray bar, *esg>UAS-D-p38b^+^*. The number of PH3-positive cells detected per midgut of 5-day-old flies was set as 1. P-values were calculated using Student's t-test.

### D-p38b MAPK is required for the age-related increase in ISC proliferation

**Figure 3.**
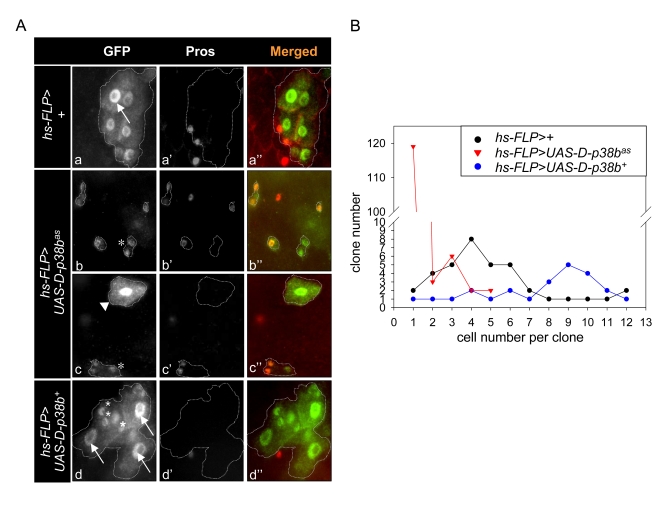
Effect of D-p38b activity on colony size in heat-shock FLP-catalysed site-specific recombination. (**A**) Lineage-marking random dividing cells by Flp-out cassette. Genotype: a-a'', *y,w,hs-FLP^122^/+;actin>y+>gal4,UAS-GFP*; b-b'', *y,w,hs-FLP^122^/UAS-D-p38b^as^;actin>y+>gal4,UAS-GFP* and *y,w,hs-FLP^122^/UAS-D-p38b^+^;actin>y+>gal4,UAS-GFP*. All clones were marked with GFP (green) and Prospero (red). An asterisk represents non-enteroendocrine small cells. Arrow head represents a transient enterocyte colony. Arrow indicates large EC cell nuclei. Original magnification is 400x. (**B**) Histograms showing colony analysis in each genotype. Genotype: closed circular, *y,w,hs-FLP^122^/+;actin>y+>gal4,UAS-GFP*; open triangle, *y,w,hs-FLP^122^/UAS-D-p38b^as^;actin>y+>gal4,UAS-GFP*, open circular, *y,w,hs-FLP^122^/UAS-D-p38b^+^*;*actin>y+>gal4,UAS-GFP*.

We previously demonstrated an age-related increase of ISC proliferation in adult *Drosophila* midgut via BrdU incorporation and PH3 cell staining [7]. Therefore, we investigated whether D-p38b MAPK is involved in the age-related increase in proliferation of ISCs. To determine activation of D-p38b in ISCs, we used *UAS-D-p38b^+^* or *UAS-D-p38b^as^* [16] and *esg-GAL4* flies, which express GAL4 and UAS-GFP in midgut ISCs and EBs [4]. Adachi-Yamada et al. reported that overexpression of *UAS-D-p38b^+^,* under control of *hs-GAL4*, increased phosphorylation and enzymatic activity of D-p38b. In addition they demonstrated that overexpression of the *UAS-D-*p38b^as ^construct, which contains the inverted cDNA, of full-length D-p38b, suppressed the ectopic tkv-induced wing phenotype [16]. Guts from 30-day-old wild-type flies, *esg>+*, showed a significant increase in the number of BrdU-labeled large and small cells compared to those from 5-day-old flies (Figure 2A, panels a and b). This is consistent with our previous study [7]. However, guts expressing D-p38b^as^ in ISCs and EBs by *esg-GAL4* showed no age-related increase in DNA synthesis in the posterior midgut (Figure 2A, panels c and d). We also observed no age-related increase in DNA synthesis in the posterior midgut from two D-p38b mutants (Figure 2A, panels e-h). Flies overexpressing D-p38b^+^ under the control of *esg-GAL4* had increased numbers of BrdU-labeled midgut cells compared to wild-type flies at 5-days-old (Supplementary Figure 1). We next analyzed the role of D-p38b MAPK in ISC division with anti-PH3 antibody, which detects only proliferating cells [4,5]. In consistent with our previous study [7], we observed an age-related increase in the number of PH3-positive cells in 5-, 30-, and 60-day-old guts in flies carrying one copy of *esg-GAL4 *(Figure 2B). However, expression of D-p38b^as^ in ISCs and EBs by *esg-GAL4* suppressed the age-related increase in cellular division of ISCs (Figure2B). We also analyzed the number of PH3-positive cells in the aged guts of two D-p38b mutants. The number of PH3-positive cells in both mutants decreased with age (Figure 2B). As expected, the guts from 5-day-old flies overexpressing D-p38b^+^ under the control of *esg-GAL4* showed a 2.6-fold increase in the number of PH3-positive cells compared to wild-type (Figure 2C). To confirm the role of D-p38b MAPK in proliferation of ISCs, we generated green fluorescent protein (GFP)-marked clones over-expressing D-p38b^+^ or D-p38b^as^ using Flp-out cassette [17] and counted the cell number per one GFP-positive cluster in the posterior midgut. The size of the colony indicates the rate of cell division of the ISCs [4]. While most colonies in the control guts contained 4-7 cells (Figure 3A and B, black circle), clones in the guts of D-p38b^as^ were composed of 1-4 cells (Figure 3A and B, red triangle). Most clones expressing ectopic D-p38b contained 9-13 cells (Figure 3A and B, blue circle). Collectively, these data indicate that D-p38b is involved in ISC division and is required for the age-related increase in ISC proliferation.

### D-p38b MAPK is involved in age-related changes of ISC and progenitor cell differentiation

**Figure 4.**
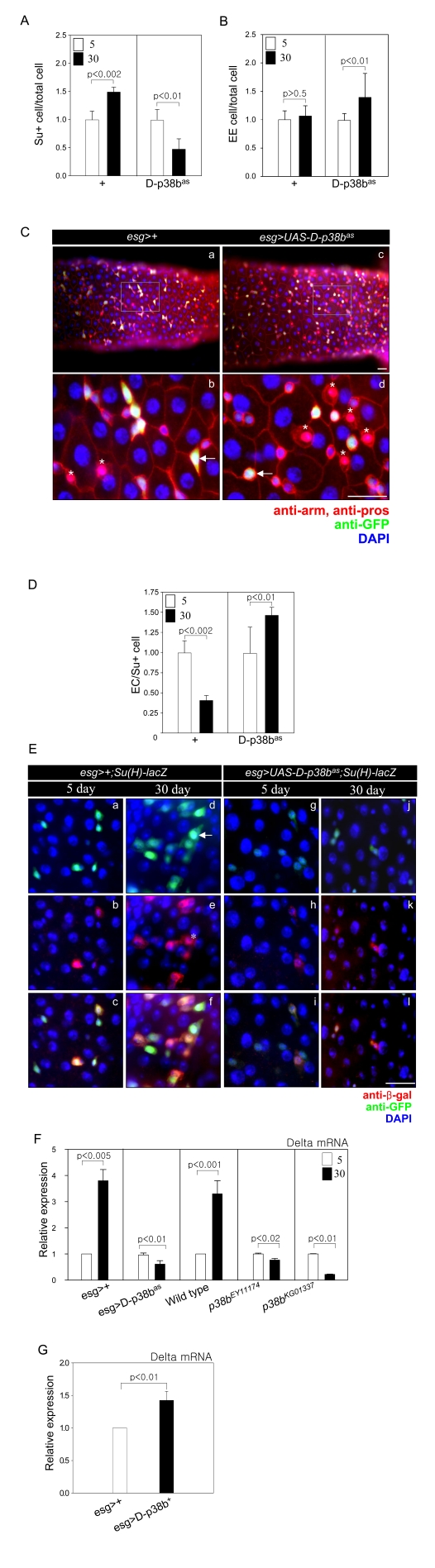
D-p38b MAPK plays a role in age-related defects in the differentiation of ISCs and progenitor cells. (**A**) Graph showing the ratio of Su(H)GBE-positive to total cells. Effects of D-p38b activity on age-related changes in the number of Su(H)GBE-positive cells in the posterior midgut. Midguts of *esg>+;Su(H)GBE-lacZ* or *esg>UAS-D-p38b^as^;Su(H)GBE-lacZ* flies were stained with DAPI, anti-β-gal and anti-GFP. The numbers of each cell type were counted in a 0.06 x 0.04 cm area of the posterior midgut. The ratio of Su(H)GBE-positive to total cells counted in the posterior midgut of 5-day-old flies was set as 1. White square, 5-day-old flies; black square, 30-day-old flies. P-values were determined using Student's t-test. (**B**) Graph showing the ratio of EE to total cells. Midguts of *esg>+;Su(H)GBE-lacZ* or *esg>UAS-D-p38b^as^;Su(H)GBE-lacZ* flies were stained with DAPI, anti-Prospero and anti-GFP. Numbers of each cell type were counted in a 0.06 x 0.04 cm area of posterior midgut. The ratio of EE to total cells counted in the posterior midgut of 5-day-old flies was set as 1. White square, 5-day-old flies; black square, 30-day-old flies. P-values were determined using Student's t-test. (**C**) Effects of D-p38b^as^ expression on ISC and EB cell morphology of esg-positive cells and differentiation of EEs. Midguts of *esg>+* (a-b) or *esg>UAS-D-p38b^as^* (c-d) flies were stained with anti-Prospero (red), anti-GFP (green) and DAPI (blue). Enlarged images, panels b and d. Scale bar, 5 μM. Arrow heads indicate esg-positive cells. Asterisks indicate EEs. (**D**) Graph showing the ratio of EC to Su(H)GBE-positive cells. Midguts of *esg>+; Su(H)GBE-lacZ* or *esg>UAS-D-p38b^as^; Su(H)GBE-lacZ* flies were stained with DAPI, anti-β-gal and anti-GFP. Numbers of each cell type were counted in a 0.06 x 0.04 cm area of posterior midgut. The ratio of EC to Su(H)GBE-positive cells counted in the posterior midgut of 5-day-old flies was set as 1. White square, 5-day-old flies; black square, 30-day-old flies. P-values were determined using Student's t-test. (**E**) Effect of D-p38b^as^ expression in ISCs and EBs on age-related accumulation of EC-like large esg- and Su(H)GBE-positive cells. The guts of 5- and 30-day-old flies were labeled with anti-β-gal and anti-GFP. (a-f) *esg>+;Su(H)GBE-lacZ*, (g-l) *esg>UAS-D-p38b^as^;Su(H)GBE-lacZ*, (m-r) *esg>UAS-D-p38b^+^; Su(H)GBE-lacZ*. (a, d, g, j, m, and p - green) anti-GFP; (b, e, h, k, n, and q - red) anti-β-gal; (c, f, I, l, o, and r) merged image. (DAPI, blue). Arrow indicates EC-like large esg-GAL4. Asterisk indicates large Su(H)GBE-positive cell. Scale bar, 5 μM. (**F**) Effect of D-p38b activity on the expression levels of Delta mRNA ISCs and EBs in adult guts. Delta mRNA was measured by quantitative RT-PCR in cDNA prepared from dissected guts from 5- and 30-day-old *esg>+*, *esg>UAS-D-p38b^as^*, wild-type, *p38b^EY11174^*, or *p38b^KG01337 ^*flies. Expression was normalized to the expression of rp49. Expression level of Delta mRNA in the midgut of 5-day-old flies was set as 1. White bar, 5-day-old flies; black bar, 30-day-old flies. P-values were determined using Student's t-test. (**G**) Effect of D-p38b^+^ on the expression of Delta in ISCs and EBs in adult gut. The level of Delta mRNA was measured by real-time RT-PCR in cDNA prepared from dissected gut from 5-day-old *esg>+ *or *esg>UAS-D-p38b^+^* flies. Expression was normalized to the expression of rp49. Expression level of Delta mRNA in midgut of 5-day-old flies was set as 1. White bar, *esg>+*; black bar, *esg>UAS-D-p38b^+^*. P-value was determined using Student's t-test.

To assess whether D-p38b MAPK is required for age-related changes in ISC and progenitor cell dif-ferentiation, we analyzed the ratio of Su(H)GBE-positive to total cells to determine the frequency of EBs differentiation to ECs. It was reported that high Su(H)GBE-positive EBs become ECs [6]. Consistent with our previous study, the number of Su(H)GBE-positive cells in the guts of control *esg>Su(H)GBE-lacZ* flies increased with age (Figure 4A) [7]. In contrast, the number of Su(H)GBE-positive cells in the guts of 30-day-old *esg>UAS-D-p38b^as^;Su(H)GBE-lacZ* flies was 0.5-fold less than that of 5-day-old flies (Figure 4A). Overexpression of D-p38b^+^ in ISCs and EBs resulted in an increased number of Su(H)GBE-positive cells compared to control flies at 5-days-old (Supplementary Figure 3A). We also examined the ratio of Prospero-positive cells, to determine the frequency of EBs differentiation to EEs. It was reported that the ratio of EEs to total cells does not change [8]. Interestingly, in guts expressing D-p38b^as^ in ISCs and EBs, the ratio of EE to total cells increased with age. A 1.48-fold increase was observed in 30-day-old compared to 5-day-old *esg>UAS-D-p38b^as^;Su(H)GBE-lacZ* flies, while of the ratio of EE to total cells did not change in control flies (Figure 4B). Esg-positive cells had a spherical cell shape in the guts of *esg>UAS-D-p38b^as^*, distinguishing them morphologically from their angularly shaped counterparts in the guts of *esg>+* (Figure 4C). We also detected that the ratio of EE to total cells in the 30-day-old posterior midgut of two D-p38b mutant flies was 2.2-fold and 2.1-fold higher than that in the 30-day-old gut of control flies (Supplementary Figure 2A and B). These results indicate that D-p38b MAPK is required for the age-related increase in ISC differentiation to ECs in the adult posterior midgut.

Next, to determine whether D-p38b MAPK is involved in the terminal differentiation defect of ECs in aged guts, we analyzed the ratio of ECs to Su(H)GBE-positive cells. In the guts of control flies, the ratio of ECs to Su(H)GBE-positive cells at 30-days-old decreased by 0.5-fold compared to that at 5-days-old. In contrast, the ratio in the guts of flies expressing D-p38b anti-sense in ISCs and EBs at 30-days-old was 1.45-fold higher than at 5-days-old (Figure 4C). The ratio of ECs to Su(H)GBE-positive cells in 5-day-old guts of *esg>UAS-D-p38b^+^;Su(H)GBE-lacZ* was slightly lower than those of control flies (Supplementary Figure 3B). These results indicate that D-p38b MAPK is involved in the defect of EC differentiation in aged guts.

We also analyzed the effects of D-p38b MAPK activation on the morphology of ISCs and EBs in the gut. We observed age-related accumulation of EC-like large esg-positive cells in the guts of control flies, *esg>Su(H)GBE-lacZ* (Figure 4D, panels a and d). Interestingly, age-related accumulation of EC-like large esg-positive cells was not detected in the guts of* esg>UAS-D-p38b^as^;Su(H)GBE-lacZ* flies (Figure 4E, panels g and j). D-p38b^+ ^overexpression in ISCs and EBs induced EC-like large esg-positive cells at 5-days- old (Supplementary Figure 3C). These results indicate that D-p38b MAPK is involved in age-related accumulation of EC-like large esg-positive cells in the posterior midgut.

We observed a 4-fold and 3.4-fold increase of Delta mRNA in 30-day-old guts compared to 5-day-old guts of control flies, *Oregon-R* and* esg>+*, respectively (Figure 4F). We examined whether D-p38b MAPK is involved in age-related modulation of Delta expression within the gut. Expectantly, Delta mRNA levels in the gut of *esg>UAS-D-p38b^as^* flies did not change with age (Figure 4F). Two D-p38b mutants also showed no age-related change in Delta expression (Figure 4F). Overexpression of D-p38b^+^ in ISCs and EBs by *esg-GAL4* increased Delta expression up to 1.5-fold compared to control flies at 5-days-old (Figure 4G). This indicates that D-p38b is involved in the regulation of Delta expression.

### D-p38b MAPK is involved in oxidative stress-induced differentiation defects

**Figure 5.**
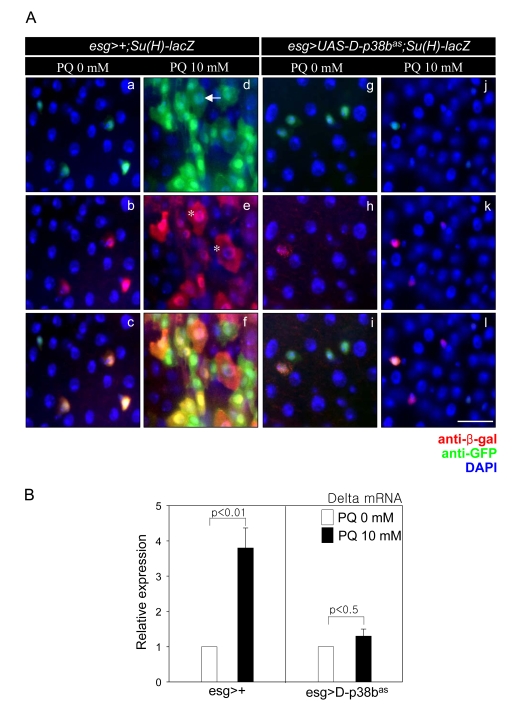
D-p38b MAPK plays a role in oxidative stress-induced aberrant Delta/ Notch signaling within the adult midgut. (**A**) Effect of D-p38b knockdown in ISCs and EBs on oxidative stress-induced accumulation of abnormal large esg- and Su(H)GBE-positive cells within the adult midgut. The midguts of 5-day-old esg>+;Su(H)GBE-lacZ (a-f) or esg>UAS-D-p38bas;Su(H)-GEB-lacZ (g-l) flies exposed to 10 mM PQ in 1% sucrose (d-f and j-l) or 1% sucrose media control (a-c and g-i) were labeled for 16 h with anti-GFP (a, d, g, and j) and anti-?-gal (b, e, h, and k - red). (c, f, i, and l) merged image. (DAPI, blue) Arrow indicates EC-like large esg-GAL4. Asterisk indicates large Su(H)GBE-positive cell. Scale bar, 5 µM. (**B**) Effect of D-p38b knockdown on oxidative stress-induced increase of Delta expression. The level of Delta mRNA was measured by quantitative RT-PCR of dissected guts from 5-day-old esg>+, esg>UAS-D-p38bas flies exposed to 10 mM PQ in 1% sucrose or 1% sucrose media control for 16 h. Expression was normalized to the expression of rp49. The level of Delta mRNA in midgut of 5-day-old flies was set as 1. White bar, 1% sucrose media; black bar, 10 mM PQ in 1% sucrose media. P-values were determined using Student′s t-test.

To examine whether D-p38b MAPK is involved in oxidative stress-induced mis-differentiation of ISCs and progenitors, we analyzed the morphology of esg- or Su(H)GBE-positive cells in guts expressing D-p38b^as^ in ISCs and EBs after paraquet (PQ) exposure. EC-like large esg- and Su(H)GBE-positive cells accumulated in the guts of 5-day-old* esg>Su(H)GBE-lacZ* flies after 10 mM PQ treatment (Figure 5A, panels a-f). In contrast, the guts of* esg>UAS-D-p38b^as^;Su(H)GBE-lacZ* flies showed no oxidative stress-induced accumulation of large esg- and Su(H)GBE-positive cells (Figure 5A, panels g-l). We also observed that expression of D-p38b^as^ in ISCs and EBs suppressed the oxidative stress-induced increase of Delta mRNA level in guts via quantitative real-time PCR (Figure 5B). These results indicate that D-p38b MAPK is involved in oxidative stress-induced mis-differentiation of ISCs and progenitors.

**Figure 6.**
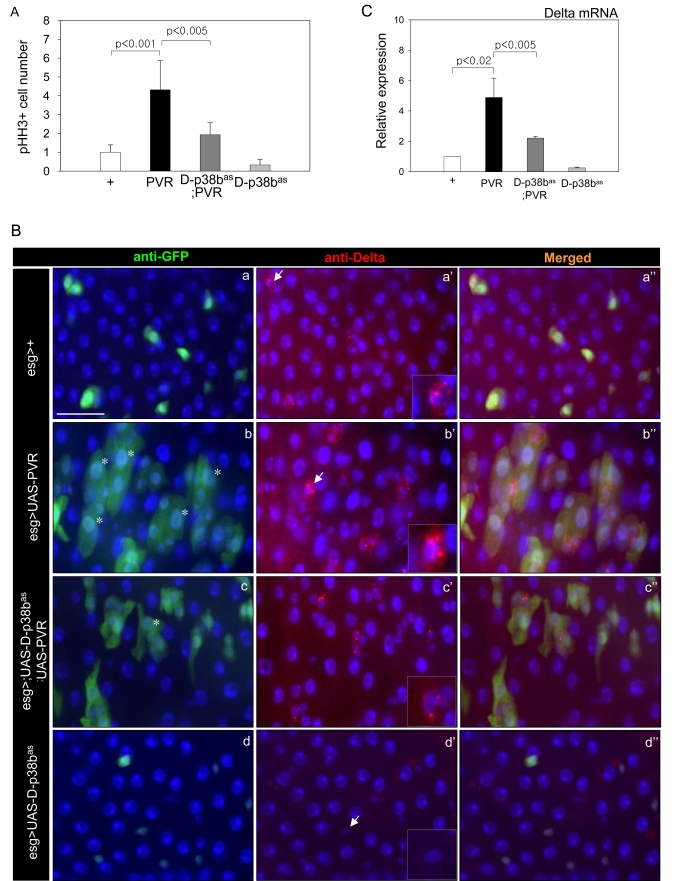
Effects of p38b MAPK knockdown in ISCs and EBs on PVR-induced phenotypes. (**A**) Effect of D-p38b knockdown in ISCs/EBs on ectopic PVR-induced ISC proliferation. The PH3-positive cells in the midgut of the 3-day-old flies were counted. White bar, *esg>+*; black bar, *esg>UAS-PVR*; dark gray bar, *esg>UAS-D-p38b^as^*;*UAS-PVR*; gray bar, *esg>UAS-D-p38b^as^*. The number of PH3-positive cells detected per midgut of *esg>+* flies was set as 1. P-values were calculated using Student's t-test. (**B**) Effect of D-p38b knockdown in ISCs and EBs on PVR-induced accumulation of large esg- and Delta-positive cells. The guts of 3-day-old flies were labeled with anti-Delta and anti-GFP. (a-a''), *esg>+*; (b-b''), *esg>UAS-PVR*; (c-c''), *esg>UAS-D-p38b^as^;UAS-PVR*; (d-d''), *esg>UAS-D-p38b^as^*. Overlay (DAPI, blue; anti-Delta, red; anti-GFP, green). Asterisk indicates EC-like large esg-positive cell. Arrow indicates Delta-positive cells. Scale bar, 5 μM. C. Effect of D-p38b knockdown in ISCs and EBs on PVR-induced Delta mRNA expression. Expression of Delta was measured by quantitative RT-PCR of dissected gut from 3-day-old flies. White bar, *esg>+*; black bar, *esg>UAS-PVR*; dark gray bar, *esg>UAS-D-p38b^as^*;*UAS-PVR*; gray bar, *esg>UAS-D-p38b^as^*. Expression was normalized to the expression of rp49. The level of Delta mRNA in the midgut of *esg>+* flies was set as 1. P-values were determined using Student's t-test.

### D-p38b is downstream of PVF2/PVR signaling in intestinal epithelium

We previously reported that PVF2/PVR signaling contributes to an age and oxidative stress-related increase in stem cell proliferation, and a defect in differentiation of ECs [7]. To investigate whether D-p38b MAPK can act downstream of PVF2/PVR signaling in the age-related changes of intestinal epithelium, we first analyzed the effect of D-p38b knockdown on the ISC division. PVR overexpression in ISCs and EBs using *esg-GAL4* driver increases ISC division [7]. However, D-p38b knockdown in ISCs and EBs partially suppressed the PVR-induced increase of PH3-positive cells (Figure 6A). These results indicate that D-p38b MAPK acts downstream of PVR signaling in midgut ISC proliferation. We next investigated whether D-p38b MAPK is related to PVR signaling in the mis-differentiation of ISCs and progenitors. We observed accumulation of EC-like large esg-positive cells in 5-day-old guts of *esg>UAS-PVR* flies (Figure 6B, panels b-b''). Interestingly, PVR-induced increase in the number of EC-like esg-positive cells was partially suppressed by D-p38b knockdown in ISCs and EBs (Figure 6B, panel c and c''). Overexpression of PVR in ISCs and EBs results in accumulation of aberrant Delta-positive cells (Figure 6B. panel b'). These cells have high Delta levels and the phenotype was partially suppressed by D-p38b knockdown in ISCs and EBs (Figure 6B, panel c'). In addition, expression of PVR in ISCs and EBs induced a 4-fold increase in Delta expression compared to control flies, which is partially suppressed by D-p38b knockdown (Figure 6C). These results indicate that D-p38b MAPK acts downstream of PVR signaling in the mis-differentiation of ISCs and progenitors.

## Discussion

In a previous study, we reported age-related changes including an increase in stem cell proliferation and a defect in stem and progenitor cell differentiation to EC. In addition we demonstrated that a PDGF/VEGF-like growth factor, PVF2/PVR, is involved in the age and oxidative stress-associated modulation in the *Drosophila* midgut [7]. Recently, Biteau et al. also reported the phenomenon of aged intestinal epithelium due to aberrant JNK activity [8]. Here, we investigated whether D-p38b MAPK activity is required for age and oxidative stress-associated changes in intestinal epithelium.

We found an age-related increase of a D-p38b reporter construct in ISCs and EBs in the adult *Drosophila* midgut. In mammals, it was reported that activated p38 MAPK was detected in the ISCs and their daughter cells after ischemia [12]. We analyzed whether D-p38b MAPK plays a role in the age-related increase of ISC proliferation in *Drosophila* midgut. Flies expressing D-p38b^as^ in ISCs and EBs and two D-p38b mutants did not show an age-related increase of ISC proliferation. Previously, we reported that PVF2/PVR signaling is involved in an age and oxidative stress-related increase in ISC proliferation [7]. Here, we showed that PVR-induced increase in ISC proliferation was suppressed by D-p38b MAPK knockdown in ISCs and EBs. These data suggest that D-p38b MAPK acts downstream of PVR signaling in ISC proliferation. However, it should be noted that D-p38b expression in ISCs and EBs partially suppressed the PVR-induced ISC proliferation.

Recently, Biteau et al. reported that JNK activity contributes to the age-associated increase in stem cell proliferation [8]. It was reported that Wg signaling, insulin receptor signaling pathway, and the JAK-STAT pathway are involved in ISC proliferation in the *Drosophila* adult midgut [9,10,17]. Jiang and Edgar reported that EGFR/RAS/ERK pathway is required for proliferation of adult midgut progenitors [18]. Therefore, cross-talk between these signaling pathways and PVR-p38 MAPK signaling in the regulation of ISC proliferation would be an interesting subject to investigate.

In a previous study, we also found that the number of EBs differentiating to ECs increased with age [7]. Ohlstein and Spradling reported that ISC within the adult midgut normally produce EB via asymmetric division. Most EBs differentiate to ECs, while a few differentiate to EEs [6]. It was reported that the ratio of EE to total cells did not change in the midgut with age [8]. These data suggest that there is an age-related increase in the number of EBs giving rise to ECs. Here, we showed that loss of D-p38 signaling suppressed the age-related increase of Su(H)GBE-positive cells and increased the ratio of EE to total cells, especially in old flies. In addition, the esg-positive cells with aberrant D-p38 signaling were spherical instead of angular shaped. Recently, Maeda *et al.* reported that knockdown of E-cad in the gut resulted in esg-positive cells with a spherical cell shape and an increase of the number of EEs [19]. Our data suggest that D-p38b MAPK is required for ISC commitment to EB differentiation to EC.

Recently, it was reported accumulation of aberrant EC-like esg-positive cells is an indicator for misdifferentiation of ECs. It was also proposed that aberrant expression of Delta in ISCs and progenitors is associated with the accumulation of large esg-positive cells [8]. In this study, expression of D-p38b^as^ in ISCs and EBs suppressed the age-related and oxidative stress-induced accumulation of EC-like esg-positive cells, and partially suppressed the mis-differentiation induced by ectopic PVR. Furthermore, loss of D-p38b MAPK signaling in ISCs and EBs suppressed age and stress-induced Delta expression, and partially suppressed PVR-induced Delta expression. These data suggest a cross-talk between PVR and D-p38b MAPK signaling in the regulation of Delta expression. Furthermore, our data suggest that D-p38b MAPK acts downstream of PVR signaling, and aberrant D-p38b MAPK signaling results in mis-differentiation in the midgut.

It was reported that in wild-type gut, ECs turnover approximately within one week [5]. Interestingly, we observed that D-p38b knockdown in ECs likely results in cell turnover longer than one week. We also detected that EC size in the guts of flies expressing D-p38b^as^ in ISCs and EBs was larger than those of controls with age (data not shown), suggesting that D-p38b MAPK may be involved in EC death. Recent studies in mammals demonstrated that p38 MAPK is involved in intestinal epithelial cell apoptosis through activation of Bax in stress conditions [20, 21].

In light of these findings, we propose that D-p38b MAPK plays a crucial role in the balance between regeneration of intestinal epithelium and proper differentiation.

## Experimental procedures

Fly stock. Fly stocks were maintained at 25 °C on standard food under an ~12 h/12 h light/dark cycle. The food consisted of 79.2% water, 1% agar, 7% cornmeal, 2% yeast, 10% sucrose, 0.3% bokinin and 0.5% propionic acid. To avoid larval overpopulation in all vials, 50-60 adult flies per vial were transferred to new food vials every 2-3 days for a period of 50-60 days or longer. We previously established the reporter transgenic flies carrying promoter region of *D-p38b* gene [15]. *UAS-D-p38b^+^* and *UAS-D-p38b^as^* were kindly provided by T. Adachi-Yamada [16]. *Su(H)GBE-lacZ* was kindly provided by Sarah Bray [22]. *UAS-PVR* was generously provided by Pernille Rørth [23]. *y w hs-Flp[122]; act>y^+^>gal-4 UAS-GFP (AyGal4)* was kindly provided by K.D. Irvine [24].**esg-GAL4** was kindly provided by the Drosophila Genetic Resource Center. *Oregon-R* was used as wild-type. *p38b^EY11174^* and *p38b^KG01337^* were established by Bellen *et al*. [25] and acquired from the Bloomington   Drosophila Stock Center.

The *UAS-D-p38b^+^ or UAS-D-p38b^as^/+;*esg-GAL,UAS-*GFP*/+*;Su(H)GBE-lacZ*/+ flies were obtained from a cross of the *UAS-D-p38b^+^ or UAS-D-p38b^as^/ y or UAS-D-p38b^as^*;+;Su(H)GBE-lacZ **/TM6 males to the *esg-GAL4,UAS-GFP*/*CyO* females. The *esg-GAL4*,*UAS-GFP*/+;*Su(H)GBE-lacZ*/+flies from a cross of the *Su(H)GBE-lacZ*/TM3 males to the *esg-GAL4,UAS-GFP*/*CyO* females were used as a control. The *UAS-D-p38b^as^/+;UAS-PVR/*esg-GAL4,UAS-GFP **flies were obtained from a cross of the *UAS-D-p38b^as^/y*;UAS-PVR/UAS-PVR** males to the *esg-GAL4,UAS-GFP*/*CyO* females.

All experiments were conducted and evidenced similar results in both females and males. The results described in this present study were obtained from the females.

Oligonucleotides. Oligonucleotide primers of rp49 and Delta were previously described [8]. All oligonucleotides were chemically synthesized.

Quantitative RT-PCR. Total RNA from the adult midguts was isolated with Trizol Reagent (Molecular Research Center, Cincinnati, OH, USA) in accordance with the protocols recommended by the manufacturer. In brief, adult midguts were dissected, chloroform was added (Sigma, St. Louis, MO, USA) and then the samples were repeatedly centrifuged at 19326 × *g* at 4 °C for 15 min. The supernatant was moved to a new microtube, isopropanol was added, and the samples were incubated at 25 °C for 15 min, centrifuged repeatedly at 19326 × *g* at 4 °C for 15 min, washed with 70% EtOH, and dried. cDNAs from the prepared mRNA extracts were synthesized. Denatured mRNA with M-MLV-RT buffer, 2.5 mM dNTP, oligo dT, 100 mM DTT and M-MLV-reverse transcriptase (Promega, Madison, WI, USA) was incubated at 42 °C for 1 h. The real-time RT-PCR products were analyzed using OpticMonitor3.

Immunochemistry. The intact adult guts were dissected, fixed at room temperature for 1 h in 4% formaldehyde (Sigma), washed with PBT [0.1% Triton X-100 in phosphate-buffered saline (PBS)], and incubated overnight with primary antibody at 4 °C. After washing and blocking [2% bovine serum albumin (BSA) in PBT], the samples were incubated for 1 h with secondary antibodies at 25 °C, washed in PBT, mounted with Vectashield (Vector Laboratories, Burlingame, CA, USA), and analyzed using a Zeiss Axioskop 2plus microscope (Carl Zeiss Inc., Gottingen, Germany). For the quantitative analysis of *esg *-, Delta- and Su(H)GBE-positive cells, EC and EE cells, images were processed in Photoshop (Adobe Systems, San Jose, CA, USA). The numbers of *esg *-, Delta- and Su(H)GBE-positive cells and ECs were counted in 0.06 × 0.04 cm area and the number of EEs were counted in 0.06 × 0.04 cm area of the posterior midgut.

BrdU labeling. BrdU staining was conducted via standard methods and modified as follows [5]. Flies were cultured on standard food supplemented with BrdU (final concentration 0.2 mg/ml) for 4 days. Flies were subsequently cultured at 25 °C and transferred to new media every 2 days. The entire guts were removed and fixed in ethanol : acetic acid (3 : 1) for 2 min and DNA was denatured by incubating tissue in 2M HCl for 10 mins, then incubated with primary antibody overnight at 4 °C. Primary antibody was removed and the samples were washed in PBT. The samples were incubated for 1 h with secondary antibodies and DAPI, washed in PBT and mounted with Vectashield.

Mosaic analysis. For clonal analysis, clones were generated by Flp-out cassette [17]. In this case, mitotic clones were conditionally induced on heat-shock treatments for flipase expression, and clones were marked by GFP. Fly crosses established and cultured at 18 °C to generate adults of the genotype *y w hs-Flp[122] /UAS-D-p38b^+^; act>y^+^>gal-4 UAS-GFP/+* or *y w hs-Flp[122] /UAS-D-p38b^as^; act>y^+^>gal-4 UAS-GFP/+* or *y w hs-Flp[122] /+; act>y^+^>gal-4 UAS-GFP/+* as a control flies. Equal numbers of adult flies were then divided into experimental and control groups. Experimental animals were subjected to between one and three 37 °C heat shocks for clone induction. After heat shock, flp-out clones were grown at 18 °C to minimize background signal from the Gal/UAS system. Midguts were examined 7 days after clone induction.

Antisera. The following primary antibodies diluted in PBT were used in these experiments: rabbit anti-β-gal (Cappel, Solon, OH, USA) 1 : 500; rabbit anti-phosphohistone H3 (Upstate, Charlottesville, VA, USA) 1 : 500; mouse anti-Armadillo, mouse anti-Prospero, mouse anti-Delta and mouse anti-BrdU (Developmental Studies Hybridoma Bank, Iowa City, IA, USA) 1 : 100; mouse anti-GFP, rabbit anti-GFP (Molecular Probes, Eugene, OR, USA) 1 : 1000. The following secondary antibodies diluted in PBT + 2% BSA were used: goat anti-rabbit FITC (Cappel) 1 : 400; goat anti-rabbit Cy3 (Jackson ImmunoResearch, West Grove, PA, USA) 1 : 400; goat anti-mouse FITC (Jackson ImmunoResearch) 1 : 400; goat anti-mouse Cy3 (Jackson Immuno-Research) 1 : 400; DAPI (Molecular Probes) 1 : 1000.

X-gal staining. The adult guts were dissected on ice and fixed for 10 min with 1% glutaraldehyde (Sigma) in 1× PBS. The samples were then washed in 1× PBS for 1 h, and stained with 0.2% X-gal (USB, Cleveland, OH, USA) in staining buffer containing 6.1 mM K_4_Fe(CN)_6_, 6.1 mM K_3_Fe(CN)_6_, 1 mM MgCl_2_, 150 mM NaCl, 10 mM Na_2_HPO_4_ and 10 mM NaH_2_PO_4_ in the dark at 25 °C during overnight.

Statistical analyses. Significance testing was conducted via Student's *t *-test.

## Supplementary figures

Supplementary Figure 1 Effect of D-p38b overexpression in ISCs and EBs on ISC DNA synthesis. Increase of BrdU incorpora-tion by ectopic D-p38b MAPK in ISCs and EBs. One-day-old esg>+ (**a**) or esg>UAS-D-p38b+ (**b**) flies were fed on 0.2 mg/ml BrdU media for 4 days and stained with anti-BrdU. Overlay (DAPI, blue; anti-BrdU, red). Scale bar, 5 μM.

Supplementary Figure 2 D-p38b MAPK plays a role in the differentiation of ISCs and progenitor cells. (**A**) Graph showing an increase in the ratio of EE to total cells in the guts of D-p38b mutants. Number of the Prospero-positive cells detected per midgut of 30-day-old flies control, p38bEY11174 or p38bKG01337 flies. The number of prospero-positive cells detected per posterior midgut of 30-day-old wild-type flies was set as 1. white bar, wild-type; black bar, p38bEY11174; gray bar, p38bKG01337. P-values were calculated using Student’s t-test and compared to each control. (**B**) Effect of p38b mutant allele expression on EE cell production. The guts of 30-day-old flies were labeled with anti-prospero and DAPI. (a), wild-type; (b), p38bEY11174; (c), p38bKG01337. (anti-prospero, red; DAPI, blue). Scale bar, 5 μM.

Supplementary Figure 3 Effect of D-p38b MAPK overexpression in ISCs and EBs on differentiation of ISC progenitor cells. (**A**) Graph showing the ratio of Su(H)GBE-positive to total cells. The 5-day-old midguts of esg>+;Su(H)GBE-lacZ or esg>UAS-D-p38b+;Su(H)GBE-lacZ flies were stained with DAPI, anti-?-gal and anti-GFP. Numbers of each cell type were counted in a 0.06 x 0.04 cm area of the posterior midgut. The ratio of Su(H)GBE-positive to total cells of 5-day-old flies was set as 1. White square, esg>+;Su(H)GBE-lacZ; black square, esg>UAS-D-p38b+;Su(H) GBE-lacZ. P-values were determined using Student’s t-test. (**B**) Graph showing the ratio of ECs to Su(H)GBE-positive cells. Midguts of five-day old esg>+;Su(H)GBE-lacZ or esg>UAS-D-p38b+;Su(H) GBE-lacZ flies were stained with DAPI, anti-β-gal and anti-GFP. Numbers of each cell type were counted in a 0.06 x 0.04 cm area of the posterior midgut. White square, esg>+; black square, esg>UAS-D-p38b+. The ratio of ECs to Su(H)GBE-positive cell of 5-day-old flies was set as 1. P-values were determined using Student’s t-test. (**C**) Effect of p38b MAPK overexpression in ISCs and EBs on the size of esg- and Su(H)GBE-positive cells. The guts of 5-day-old flies were labeled with anti-β-gal and anti-GFP. (a-c) esg>+;Su(H)GBE- lacZ, (d-f) esg>UAS-D-p38b+;Su(H)GBE-lacZ. a and d, anti-GFP; b and e, anti-β-gal; c and f, merged image. (DAPI, blue; anti-?-gal, red; anti-GFP, green). Scale bar, 5 μM.
