# Supplementary figures and images for: The role of p38b MAPK in age-related modulation of intestinal stem cell proliferation and differentiation in Drosophila
Source: Aging (Albany NY). 2009 May 21;1(7):637–51. doi: 10.18632/aging.100054 (PMC2806044; doi:10.18632/aging.100054)

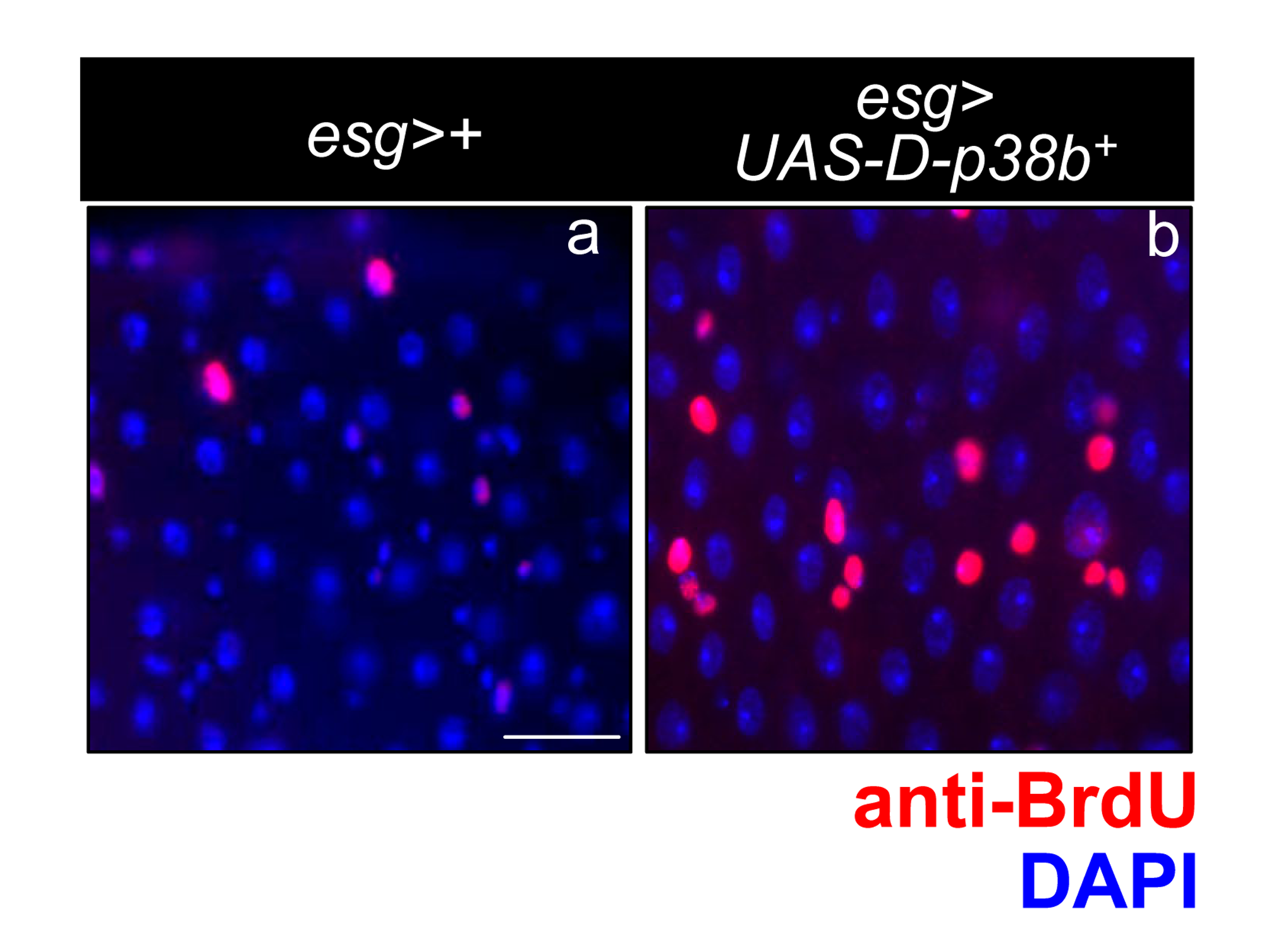

Supplement: Supplementary Figure 1 — Increase of BrdU incorpora-tion by ectopic D-p38b MAPK in ISCs and EBs. One-day-old esg>+ (a) or esg>UAS-D-p38b+ (b) flies were fed on 0.2 mg/ml BrdU media for 4 days and stained with anti-BrdU. Overlay (DAPI, blue; anti-BrdU, red). Scale bar, 5 μM. [file aging-01-637-s001.tif]

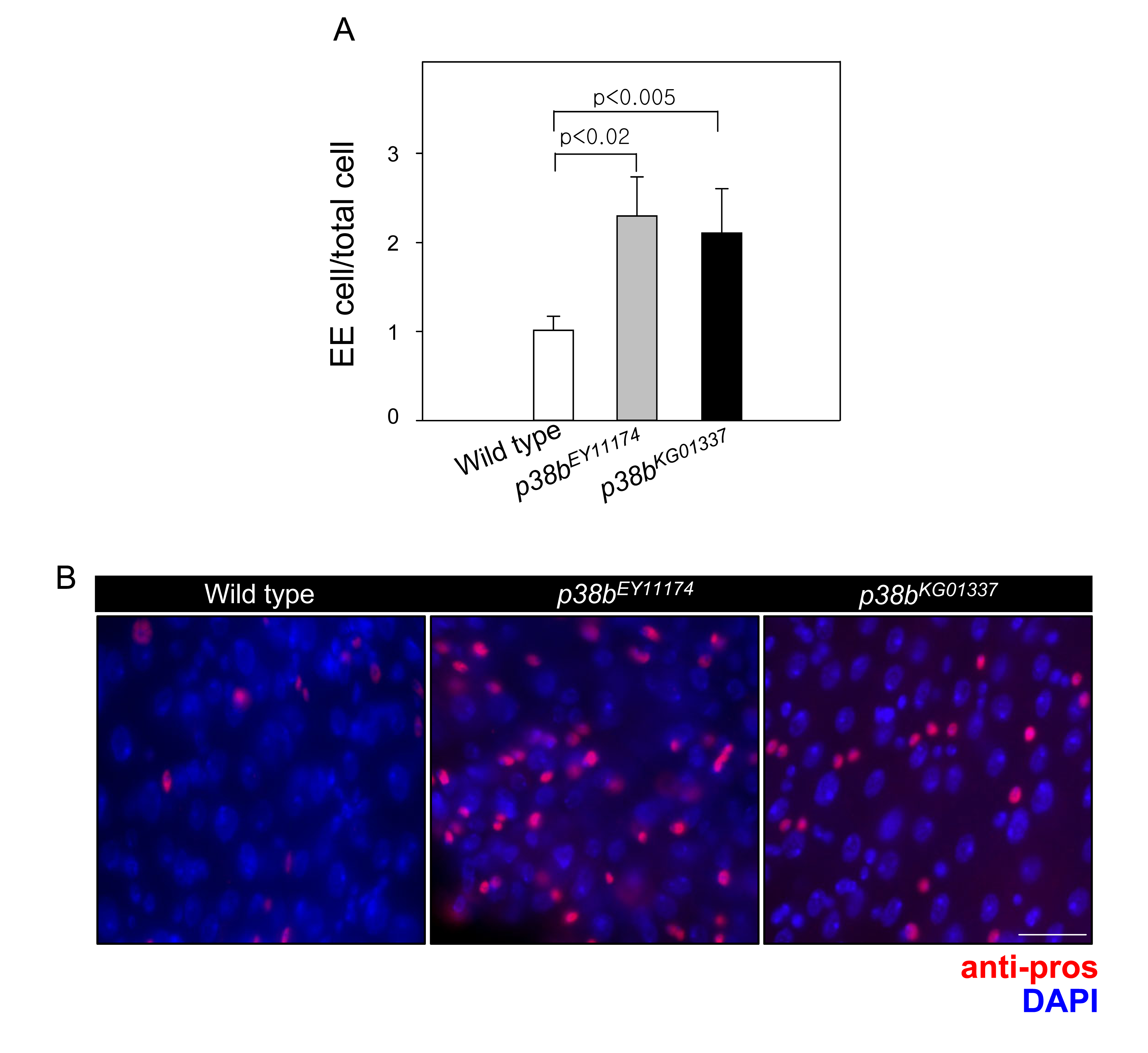

Supplement: Supplementary Figure 2 — (A) Graph showing an increase in the ratio of EE to total cells in the guts of D-p38b mutants. Number of the Prospero-positive cells detected per midgut of 30-day-old flies control, p38bEY11174 or p38bKG01337 flies. The number of prospero-positive cells detected per posterior midgut of 30-day-old wild-type flies was set as 1. white bar, wild-type; black bar, p38bEY11174; gray bar, p38bKG01337. P-values were calculated using Student’s t-test and compared to each control. (B) Effect of p38b mutant allele expression on EE cell production. The guts of 30-day-old flies were labeled with anti-prospero and DAPI. (a), wild-type; (b), p38bEY11174; (c), p38bKG01337. (anti-prospero, red; DAPI, blue). Scale bar, 5 μM. [file aging-01-637-s002.tif]

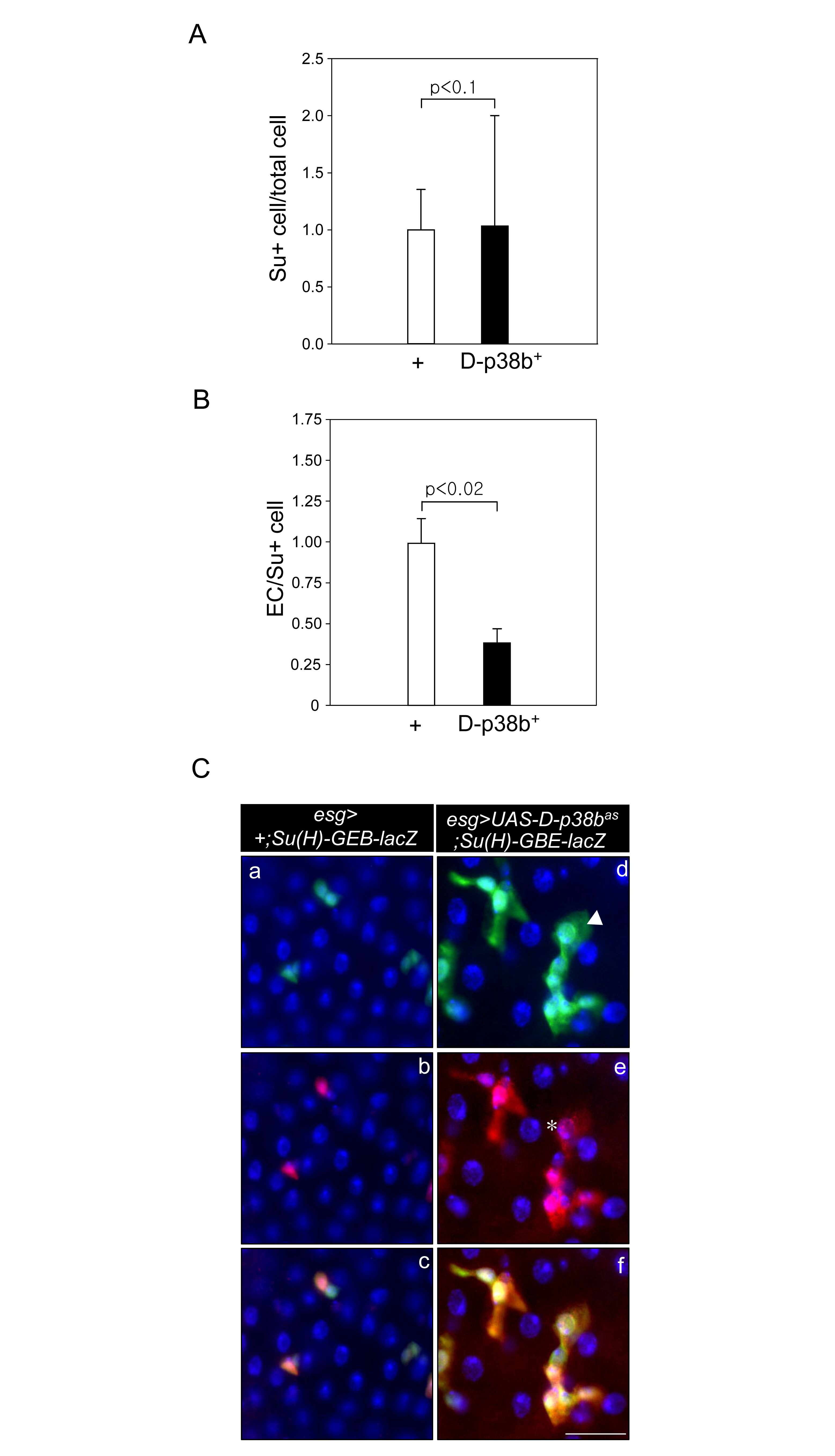

Supplement: Supplementary Figure 3 — (A) Graph showing the ratio of Su(H)GBE-positive to total cells. The 5-day-old midguts of esg>+;Su(H)GBE-lacZ or esg>UAS-D-p38b+;Su(H)GBE-lacZ flies were stained with DAPI, anti-?-gal and anti-GFP. Numbers of each cell type were counted in a 0.06 x 0.04 cm area of the posterior midgut. The ratio of Su(H)GBE-positive to total cells of 5-day-old flies was set as 1. White square, esg>+;Su(H)GBE-lacZ; black square, esg>UAS-D-p38b+;Su(H) GBE-lacZ. P-values were determined using Student’s t-test. (B) Graph showing the ratio of ECs to Su(H)GBE-positive cells. Midguts of five-day old esg>+;Su(H)GBE-lacZ or esg>UAS-D-p38b+;Su(H) GBE-lacZ flies were stained with DAPI, anti-β-gal and anti-GFP. Numbers of each cell type were counted in a 0.06 x 0.04 cm area of the posterior midgut. White square, esg>+; black square, esg>UAS-D-p38b+. The ratio of ECs to Su(H)GBE-positive cell of 5-day-old flies was set as 1. P-values were determined using Student’s t-test. (C) Effect of p38b MAPK overexpression in ISCs and EBs on the size of esg- and Su(H)GBE-positive cells. The guts of 5-day-old flies were labeled with anti-β-gal and anti-GFP. (a-c) esg>+;Su(H)GBE- lacZ, (d-f) esg>UAS-D-p38b+;Su(H)GBE-lacZ. a and d, anti-GFP; b and e, anti-β-gal; c and f, merged image. (DAPI, blue; anti-?-gal, red; anti-GFP, green). Scale bar, 5 μM. [file aging-01-637-s003.tif]
